# Transcranial Direct Current Stimulation at 4 mA Induces Greater Leg Muscle Fatigability in Women Compared to Men

**DOI:** 10.3390/brainsci10040244

**Published:** 2020-04-21

**Authors:** Craig D. Workman, Alexandra C. Fietsam, Thorsten Rudroff

**Affiliations:** 1 Department of Health and Human Physiology, University of Iowa, Iowa City, IA 52242, USA; craig-workman@uiowa.edu (C.D.W.); alexandra-fietsam@uiowa.edu (A.C.F.); 2 Department of Neurology, University of Iowa Hospitals and Clinics, Iowa City, IA 52242, USA

**Keywords:** sex differences, stimulation, fatigue, fatigability

## Abstract

Transcranial direct current stimulation (tDCS) has previously shown different cortical excitability and neuropsychological effects between women and men. However, the sex-specific effects of tDCS on leg muscle fatigability has not been investigated. The purpose of this study was to determine the effects of a single session of 2 mA and 4 mA primary motor cortex tDCS on leg muscle fatigability in healthy young men and women in a crossover design. Twenty participants (women = 10) completed isokinetic fatigue testing (40 maximal reps, 120°/s) of the knee extensors and flexors in conjunction with sham, 2 mA, and 4 mA tDCS in a double-blind, randomized design. The fatigue index from each condition was calculated. Women had significantly greater knee extensor fatigability in the 4 mA condition compared to men (57.8 ± 6.8% versus 44.1 ± 18.4%; *p* = 0.041, **d** = 0.99). This study provides additional evidence that responses to tDCS may be sex-specific and highlights the necessity of accounting and powering for sex differences in future investigations.

## 1. Introduction

Transcranial direct current stimulation (tDCS) is a non-invasive method of modulating the excitability of the cortex. tDCS applied over the motor cortex is presumed to be polarity-dependent, with brain areas under the anode increasing excitation and areas under the cathode decreasing excitation [1]. A current impediment to research of the augmentation of brain function with tDCS is the vast diversity and inconsistency in outcome results of tDCS studies. Investigations with similar designs often report different results, and study-specific parameters and tasks can only partially account for these discrepancies. Thus, despite the promising potential of tDCS for patient recovery and treatment [2], such inconsistencies and recent contrary findings [3,4] have caused some to doubt the efficacy tDCS.

There is growing evidence suggesting that neuroplastic changes from tDCS might be influenced by sex-specific variables. Kuo et al. [5] reported sex differences in the primary motor cortex after short-duration and long-duration tDCS. In their study, women had prolonged after-effects of cathodal stimulation (at 0, 10, 20, 30, 60, and 90 min post-stimulation), and men exhibited stronger anodal after-effects (at 90 min post-stimulation). Other studies have also suggested potential sex-mediated differences in tDCS responses, which are attributed to variations in skull structure, such as cortical bone density and cancellous bone thickness [6], and background hormone levels [7,8]. In addition, some studies have reported sex-specific cognitive effects from tDCS applied to different brain regions. For example, differences between women and men from stimulation of the dorsolateral prefrontal cortex for verbal working memory [9], the left parietal cortex for visual spatial attention [10], the bilateral temporal cortex for somatosensory integration [11], and the bilateral superior temporal cortex for facial expression recognition [12] have all been reported. Interestingly, no studies to date have investigated sex-related differences in tDCS responses to motor tasks, such as performance fatigability.

Fatigue is a common outcome measure in tDCS research and is defined as “the decrease in physical and/or mental performance that results from changes in central, psychological, and/or peripheral factors” [13]. In addition, performance fatigability is the magnitude or rate of change in performance over a given time relative to a reference value. The effects of tDCS on performance fatigability in healthy participants [14,15,16] and in people with neurological impairments [17,18,19,20,21,22] has been previously investigated and has yielded ambiguous findings. The mixed results might be from a lack of standardized protocols and inconsistent definitions of fatigue, which makes comparison of fatigue outcomes between studies more challenging. Nevertheless, a majority have used isometric contractions, tDCS intensities less ≤ 2 mA, and administered stimulation before a given task. Only two studies [14,15] applied different intensities of tDCS (2 mA and 4 mA) during an isokinetic fatigue task in healthy young adults. Surprisingly, the authors found that both lower and higher intensity tDCS resulted in greater leg muscle fatigability compared to sham. However, all of the performance fatigue studies described above included participant groups with a mixture of men and women, and none treated sex as a discriminating factor.

Considering that previous studies propose potentially conflicting findings, e.g., men might receive more current at the cortex than women [6] while women might have more pronounced tDCS effects than men [10,12], investigation of differences in tDCS responses between men and women is a meaningful issue to explore. Such studies will help uncover the optimal tDCS intensities (or range of intensities) unique to each sex and avoid exposing participants to higher current densities than may be required, which would help avoid problems with unacceptable scalp sensations and controlling for placebo effects. Therefore, the primary aim of the present study was to address this topic by determining the effects of 2 mA and 4 mA tDCS on leg muscle fatigability in healthy young men and women, using objective performance fatigability data (i.e., peak torque, [23]). It was hypothesized that tDCS at either intensity would induce sex-related differences in the performance of a repetitive isokinetic fatigue task.

## 2. Materials and Methods

### 2.1. Participants

Twenty healthy young adults were recruited (women = 10; mean ± SD, age = 24.6 ± 3.8 years, height = 171.1 ± 11.1 cm, weight = 71.7 ± 14.0 kg). Inclusion criteria included the following: (1) young adult (between 18 and 30 years old); (2) physically active (perform at least 30 min of moderate intensity physical activity on at least 3 days of the week for the previous 3 months); (3) right-side dominant; (4) no chronic neurological, psychiatric, or medical conditions; and (5) not taking any psychoactive medications. Exclusion criteria included the following: (1) pregnancy; (2) holes or fissures in the skull; and (3) metal objects or other devices implanted in the skull (e.g., metal plate). This study was approved by the Institutional Review Board at the University and Iowa in accordance with the Declaration of Helsinki, and all participants provided written informed consent before participating.

### 2.2. Experimental Protocol

Participants completed four experimental sessions spaced 5–8 days apart for this double-blind, randomized, sham-controlled study. During the first session, right-side dominance was objectively tested with isokinetic maximal voluntary contraction testing of the knee extensors and flexors to help avoid the potential for functional brain morphology differences between right-dominant and left-dominant participants [24]. Only participants who were determined to be right-side dominant were retained for participation in the study. After strength testing, and in order to become familiarized with the isokinetic fatigue task (FT), participants performed the right leg fatigue task (R-FT) and left leg fatigue task (L-FT) with a short (<2 min) interval between each task. The R-FT was always completed first in the familiarization and subsequent tDCS sessions. During sessions 2–4 (tDCS sessions), participants were randomly administered one of three tDCS intensities (sham, 2 mA, and 4 mA) during the R-FT and L-FT.

### 2.3. Isokinetic Strength Testing

The isokinetic tests (strength and fatigue) were delivered with an isokinetic dynamometer (HUMAC NORM, CSMi, Stoughton, MA, USA). Strength testing began with a submaximal warm-up of the knee extensors and flexors consisting of 15 concentric/concentric repetitions at 60°/s. After a short rest (≥30 s), the participants performed five sets of one repetition of maximal effort knee extension and flexion (concentric/concentric, 60°/s) on the right leg with ≥30 s of rest between each set. After a 2 min rest, participants repeated the strength testing on the left leg. During the test, administrators provided verbal encouragement to the participants to promote a maximal effort performance. Dominance was verified by examining the highest peak torque from the five maximal efforts on each leg.

### 2.4. Isokinetic Fatigue Testing

The FT commenced with the same submaximal warm-up as the strength test (15 reps, concentric/concentric, 60°/s) and consisted of 40 continuous concentric/concentric maximal contractions of the knee extensors and flexors at 120°/s [25]. This FT protocol has been well established in both healthy people and people with neurological disorders [14,15,26,27,28,29,30]. At minute 15 of the tDCS protocol (see “tDCS sessions” below), the R-FT was performed, followed by the L-FT. Including the transition time between the R-FT and L-FT, the total time for the FT was 4.5–5 min. Verbal and visual (i.e., from test administrators and bar graph of work achieved, respectively) feedback was given to participants to encourage them to achieve their maximal effort during each contraction.

### 2.5. tDCS Sessions

**Figure 1:**
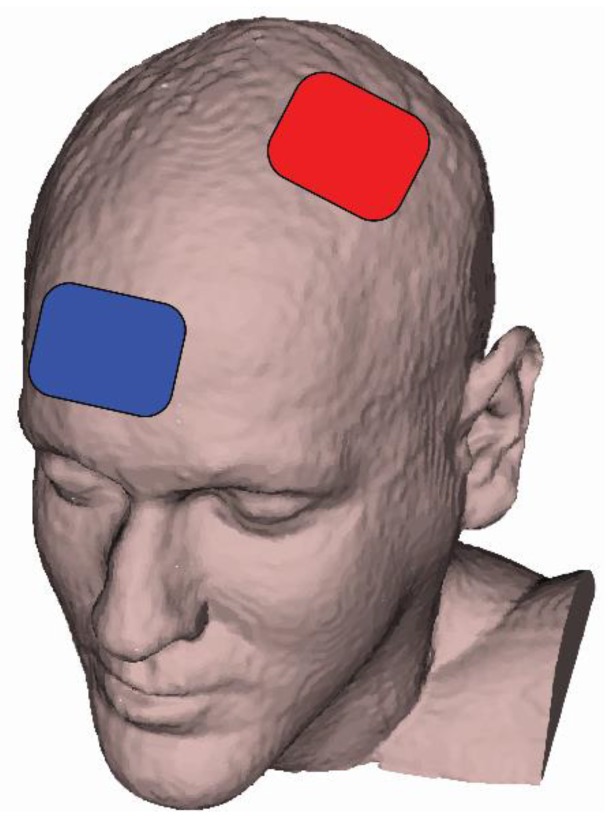
Locations of the anode (red) and cathode (blue) for the transcranial direct current stimulation.

tDCS was delivered with a battery powered 1x1 tDCS Low-Intensity Stimulator (Model 1300A, Soterix Medical Inc., New York, NY, USA) through two carbon electrodes inserted into 5 cm × 7 cm EASYpad sponges (35 cm^2^ surface area; Soterix Medical Inc., New York, NY, USA). The electrodes were soaked in 10–15 mL of 0.09% NaCL saline and held in place using an EASYstrap (Soterix Medical Inc., New York, NY, USA). The current density was 0.06 mA/cm^2^ for the 2 mA intensity and 0.11 mA/cm^2^ for the 4 mA intensity. The anode was placed over C3, using the 10-20 EEG placement convention [31], and the cathode was placed over the supraorbital area on the contralateral side (Figure 1).

This montage was chosen to maximize motor performance by targeting the dominant M1 [32]. Similar to previous tDCS studies targeting unilateral M1 leg areas [33,34], the anode bordered or covered the center of the skull (Cz). Therefore, the anode also covered the leg area of the dominant M1, which is located in the longitudinal fissure [34]. During active tDCS conditions, the stimulation ramped-up over a 30 s period and then remained at the target intensity for 20 min before ramping back down to 0 mA over 30 s. During sham tDCS, the stimulator performed a 30 s ramp-up to 2 mA followed by an immediate 30 s ramp-down to 0 mA in order simulate stimulation-related sensations and maintain condition blinding [35]. After this initial ramp-up and immediate ramp-down, the tDCS intensity remained at 0 mA. To ensure good contact quality between the anode and cathode, a “Pre-Stim Tickle” function was activated for 30 s at 1 mA. This function provides feedback about how well the anode and cathode were connected; adjustments to the electrodes (ensuring they were adequately soaked and securely placed) were made as necessary. To ensure the same electrode placement during subsequent tDCS sessions, the location of the electrodes on the EASYstrap (which has distance markings similar to a ruler) was recorded for each participant during the first tDCS session. tDCS was delivered while participants were seated in the dynamometer chair. At minute 15 of stimulation, the R-FT and the L-FT were performed over the final 5 min of the tDCS condition. Stimulation before performing a task may activate neuronal populations in a non-specific way [36]. In addition, although most have stimulated before a task [37], some motor and cognitive learning studies [38,39,40,41] have demonstrated that tDCS during a task may theoretically enhance endogenous signals during task execution and therefore benefit performance.

After the completion of the FT and tDCS, participants were asked to describe the sensations they experienced from stimulation (e.g., itching, tingling, burning, etc.) and to rate stimulation severity on a 10-point (1 = “barely perceptible” and 10 = “most I could possible stand”) Likert-type scale [14,15,42]. Participants were also asked to guess which stimulation intensity (sham, 2 mA, or 4 mA) they received in their session. Responses were recorded, but feedback about guesses was not provided until the end of the final session, at which time experimenter blinding for that subject was also broken.

### 2.6. Data Analysis

**Figure 2:**
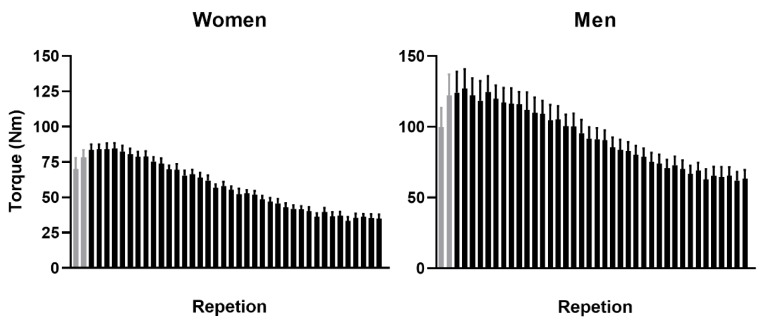
Peak torque of the right knee extensors for women (*n* = 10; left) and men (*n* = 10, right). The bars represent the mean ± SEM of the maximum torque achieved during the repetition. Note that the first two repetitions (light gray) were considered as adaptations and were not included in the fatigue index calculation.

To examine the effect of different tDCS intensities on leg muscle fatigue, a torque-derived fatigue index (FI-T) was calculated for both the knee extensors and flexors on each leg. The FI-T was computed using the peak torque from each repetition of the FT as follows: ([mean of the first five repetitions – mean of last five repetitions]/[mean of first five repetitions]) x 100 [14,15,26,27,29,30]. The first two repetitions of each FT were considered as adaptations to the FT and were excluded from the FI-T calculation [14,15] (Figure 2). The stimulation sensation severity reports were averaged for similar sensations to determine tDCS tolerability, and the percent of correct tDCS condition guesses determined blinding integrity.

### 2.7. Statistical Analysis

Differences between the knee extensors and flexors were expected *a priori* [43] and were not included in the analysis. The FI-T data for the right knee extensors and right knee flexors were investigated with a repeated-measures ANOVA, with stimulation (sham, 2 mA, and 4 mA) as a within-subjects factor and sex (male, female) as a between-subjects factor. Post-hoc pairwise testing and effect sizes (Cohen’s **d**) clarified significant main and interaction effects. The assumptions for all statistical tests (e.g., normality, linearity, sphericity) were investigated via histograms, skewness and kurtosis statistics, Q-Q plots, the Shapiro–Wilk test, and Mauchly’s Test of Sphericity. Significance was accepted at *p* < 0.05, after a Bonferroni correction. Analyses were performed using SPSS 25 (IMB Corp, Armonk, NY, USA).

## 3. Results

**Figure 3:**
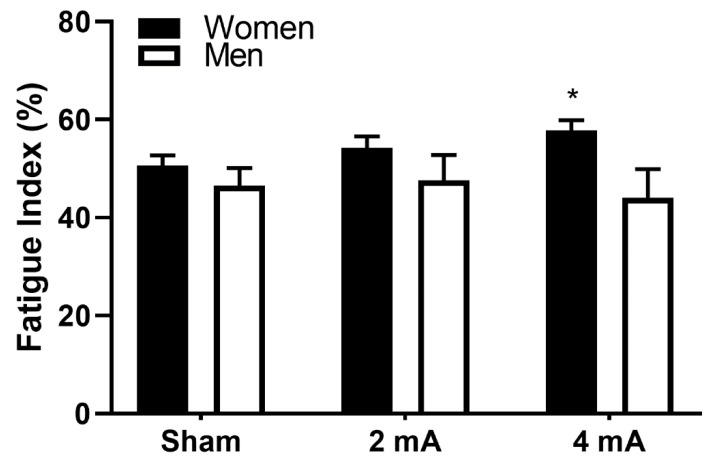
Fatigue index of the right knee extensors, stratified by transcranial direct current stimulation (tDCS) condition and sex. Data are mean ± SEM. * indicates 4 mA condition in women significantly greater than the 4 mA condition in men.

All participants completed all testing sessions, and there were no incomplete datasets. All variables met the assumptions for the statistical tests, and no adjustments were made. Data in the text are mean ± SD and mean ± SEM in the figures. The results of the repeated measures ANOVA indicated a significant stimulation X sex interaction for the right knee extensors (*p* = 0.027), but no significant stimulation or sex main effects (*p* = 0.302 and *p* = 0.123, respectively). Pairwise testing indicated that the women had significantly greater knee extensor fatigability in the 4 mA condition compared to men (57.8 ± 6.8% versus 44.1 ± 18.4%; *p* = 0.041, **d** = 0.99; Figure 3). There was also a trend toward a significantly greater fatigability in women in the 4 mA condition compared to the sham condition (57.8 ± 6.8% versus 50.6 ± 6.7%, *p* = 0.069, **d** = 1.07).

The tolerability reports and blinding integrity for men and women are reported in Table 1. Overall, the tDCS was well tolerated by both participant groups and were mild to moderate in severity. As for stimulation blinding, a majority of women and men correctly guessed sham (60% and 70%, respectively) and a nearly equivalent number of women and men correctly guessed the 2 mA and 4 mA conditions (50% and 60%; 40% and 50%, respectively).

## 4. Discussion

The present study examined, for the first time, the interaction between sex and isokinetic FT response to tDCS. It was hypothesized that either tDCS intensity would induce sex-related differences in the performance of the fatiguing isokinetic task. The results support this hypothesis. The men exhibited similar fatigability of the right knee extensors as women in the sham and 2 mA conditions but were significantly less fatigable (lower FI) than women in the 4 mA condition. In addition, the women had a trend toward significance between sham and 4 mA, but there were no differences between any tDCS intensities in the men. This study adds to previous findings [14,15], which also showed increased fatigability in young, healthy adults (men and women) from 2 mA and 4 mA tDCS during a similar isokinetic FT. In the current study, only 4 mA tDCS showed a trend of being significantly different from sham in women, but there was no effect at sham, 2 mA, and 4 mA on fatigability in men. The increased fatigability of the right knee extensors in women in the 4 mA tDCS condition might have resulted from altered motor unit recruitment/discharge rate or cortical hyperexcitability, as previously described [15].

Possible explanations for these different observed responses to tDCS might be (1) variations in skull structure/composition between men and women, such as cortical bone density and cancellous bone thickness [6], and (2) disparate background hormone levels common between men and women [7,8]. Russell et al. [6] used Magnetic Resonance Imaging (MRI) informed electrical current stimulation modeling to estimate the current intensity received at the cortex of men and women from 0.5 mA, 1 mA, and 2 mA tDCS. Their results revealed significant sex differences, with men receiving approximately 45% more cortical current than women. The large differences in frontal-parietal current density values were particularly significant. The authors attributed their findings to the different bone structures observed between the women and the men. Specifically, the men had thicker parietal skulls, which were primarily composed of cancellous bone, and the women had thinner parietal skulls, containing mostly cortical bone. Given that cortical bone is denser that cancellous bone, the authors concluded that a higher composition of cortical bone resulted in less current arriving at the cortex. However, the results of the present study contradict those of Russel et al. [6] and might be explained by the differences in age between of the two groups of participants. In the present study, the mean ± SD age was 24 ± 3.5 years in men (range = 20–30 years) and 25 ± 4.3 years in women (range = 19–30 years). In Russell et al. [6], the mean ± SD age for men was 53.0 ± 11.5 years (range of 34–68 years) and 50.5 ± 14.3 years (range of 21–75 years) for women. Bone density, including the skull, deteriorates with age in women and remains consistent in men over their lifetime [44]. Therefore, considering the results of the present study, it is suggested that skull bone density does not completely explain differences in received brain current between men and women. Interestingly, there was a trend toward significant stimulation condition differences in the 4 mA condition compared to sham (*p* = 0.069) in women, while there were no other differences observed in women or in men. This lack of significance might be due to the small number of participants (*n* = 10 in each group), but it may also strengthen the argument that higher intensities, e.g., ≥ 4 mA as suggested by Vöröslakos et al. [4], might be required for sufficient current to reach the brain [4,15,22,45]. Vöröslakos et al. [4] showed that only a small fraction of the transcranial current might reach the brain and up to 75% might be lost at the scalp, subcutaneous tissue, muscle, and skull. These tissues serve as an effective shunt and may result in at least a 50% reduction of current intensity in the soft tissues (e.g., scalp and subcutaneous tissues) and a 10%–25% (depending on skull thickness) reduction from the serial resistance of the skull. Nevertheless, exploration of the higher intensities to get effective current past the electrical shunts, 4–6 mA as indicated by their findings [4], should only be explored with extreme caution and sufficient justification. On the contrary, preliminary evidence from living patients implanted with deep brain stimulation electrodes (*n* = 3) indicated that dose-dependent tDCS-induced electrical fields were present at the basal ganglia [46].

The finding of no difference between men and women in leg muscle fatigability during an isokinetic FT, especially in the sham condition, is interesting as it diverges from the commonly reported fatigue-resistance advantage demonstrated by women. Over the past several decades, there has been mounting evidence that women are more resistant to muscle fatigue than men (for review see [47]). However, there are also some contrary reports of comparable fatigue between men and women [48,49]. Nevertheless, sex differences in leg muscle fatigability with the current experimental protocol might have been expected. Although it is difficult to ascertain the precise reason for our null findings, the lack of sex differences is likely related to the concept that the prevailing mechanisms that impair performance vary with the characteristics of the task being performed (task specificity) [50,51]. Furthermore, one study found sex differences in fatigability for repeated slow-velocity contractions [52], but these are tempered when the repeated contractions are performed at a relatively high velocity [53], similar to the FT in the present study. A possible explanation for the diminished sex difference during fast-velocity contractions may involve the distinct energy utilization of the different muscle fiber types (the composition of which differs between men and women) predominantly recruited at slow and fast velocities. Relative to isometric contractions, larger increases in energy utilization were seen for ‘slow’ muscles compared with ‘fast’ muscles during maximal-velocity contractions of animal muscle [54]. Extrapolating this finding to humans, it can be reasonably assumed that the greater fatigue resistance of women compared with men during slow contractions would be diminished during fast-velocity contractions involving smaller work-to-rest ratios.

Finally, both intensities, 2 mA and 4 mA, were well tolerated by men and women, and these tolerability reports are similar to previous findings [14,15]. A few participants reported moderate sensations (severity = 4–6), but these occurred in each tDCS condition, including sham. Importantly, all of the participants reported that these sensations were only present during the stimulation period and were resolved within minutes after stimulation ended. Nevertheless, future studies using stimulation intensities >2 mA warrant considerate application and consistent communication with participants before, during, and after tDCS application. For the stimulation blinding, a majority correctly guessed sham (women = 60%, men => 70%), while approximately half correctly guessed 2 mA (women = 50%, men = 60%) and 4 mA (women = 40%, men = 50%). However, an equal number of participants incorrectly guessed 2 mA (women = 40%, men = 40%) for the 4 mA condition. Thus, only 20% of women and 10% of men incorrectly guessed sham for the 4 mA condition. These data indicate that participant blinding at higher intensities may not be as feasible as more moderate intensities (i.e., ≤2 mA) [55,56]. One possible solution to improve blinding integrity at higher intensities and over repeated sessions would be to slightly alter the duration of the stimulation during the sham condition [57,58]. This might be accomplished by ramping up over 30 s, maintaining the stimulation for 30 s, and then ramping down over 30 s; this sham paradigm has been found to achieve high levels of blinding [58].

There are some limitations in this study. It is possible that different hormone levels affected the effectiveness of tDCS in this study. Previous studies have shown that ovarian hormones, such as estrogen, enhanced neural excitability by modulating the activity of neurotransmitter receptors (GABA-A and N-methyl-D-aspartate [NMDA] receptors), and indirectly, by modulating ion channels (fast non-genomic action) [59,60,61,62]. Inghilleri et al. [63] reported that high estrogen levels in the late follicular phase of the menstrual cycle increased cortical excitability similar to men, as measured by increases in motor evoked potential size after excitability-enhancing repetitive transcranial motor stimulation (rTMS). On the other hand, during menstrual phases with lower estrogen levels, excitability enhancements in women were blunted. Hormone levels and menstrual cycles of the women in this study were not investigated and may have affected the results. Thus, it is possible that the respective sex differences in the present study were influenced by potentially different hormone levels in women. The relatively small number of men and women should also be considered when generalizing the results to other populations or studies. However, despite the small number of subjects (*n* = 10) in each group, the effects sizes of the significant and trending results were large (**d** = 0.99 and 1.07, respectively) and strengthen the validity of these preliminary findings. In addition, a measure of cortical excitability was not available or obtained, and the excitability changes from either tDCS intensity (2 mA, 4 mA) is therefore unclear. Maintaining participant blinding integrity in repeated-session tDCS studies (when participants experience multiple tDCS conditions) is a constant limitation for repeated-measure study designs [64], and challenges with blinding might be greater at higher intensities (>2 mA). Sensation blinding might also be assessed by determining sensations experienced at different times during the stimulation period [45] or by changing the ‘target’ stimulation in the sham condition to match or exceed the highest used intensity. In this study, a common sham stimulation paradigm was used, but alternate methods, such as those described above [58], might improve blinding effectiveness.

Future studies should continue evaluating sex differences in tDCS responses, especially for motor performances. Furthermore, prospective investigations are needed to elucidate the specific role of sex hormones, in both women and in men, on cortical neuroplasticity during tDCS experiments. In addition, investigations that include both men and women should consider the potential impact that sex differences may pose to study outcomes and control or power for these in their analyses. Considering the purported influences of skull structure and hormone levels on tDCS effectiveness, and that bone structure and hormones change differently in men and women with aging, more studies are also needed to clarify the roles and interactions of sex and age on stimulation-related outcomes. The discovery of optimal stimulation parameters specific to each sex (e.g., lower intensity for men, higher intensity for women, or vice versa) might represent an important first step toward customized tDCS parameters for individual participants. Lastly, in addition to the unclear contribution of sex differences in tDCS studies, remaining open questions such as the optimal timing of tDCS (before or during), the effects of stimulation brain location (e.g., M1 vs. prefrontal areas), and the effects of aging need to be investigated. The underlying mechanisms of tDCS should be also addressed with transcranial magnetic stimulation (TMS), electromyography (EMG), and neuroimaging techniques, such as positron emission tomography (PET) and functional magnetic resonance imaging (fMRI).

## 5. Conclusions

Four mA tDCS induced greater leg muscle fatigability in women than in men, potentially from sex-related differences in skull structure (bone thickness and density) and hormone levels. No sex differences in fatigability (i.e., in the sham condition) were observed, which was likely due to task specificity and the high contraction velocity of the FT. Both tDCS intensities (2 mA and 4 mA) were generally well tolerated by men and women, and most sensation severities were similar between the tDCS conditions. The results of this study indicate that sex differences should be considered in tDCS studies, and that sex-specific adjustments to stimulation intensities should be investigated. However, if such a strategy is applied, men or women might be exposed to higher current densities, which may present difficulties in controlling for blinding and placebo effects. Ideally, study designs should consider the influence of sex, skull density, and hormones, in addition to electrode size and location, because each of these variables may influence the results. These variables should be carefully weighed when designing or interpreting outcomes and may have important consequences in clinical practice.

## Figures and Tables

**Table 1:** Stimulation sensation reports and blinding results from transcranial direct current stimulation.

	Sham		2 mA		4 mA	
	Women	Men	Women	Men	Women	Men
**Sensation**						
Tingling	2.0 ± 0.0 (*n* = 1)	1.8 ± 0.8 (*n* = 5)	2.8 ± 1.0 (*n* = 4)	1.8 ± 1.1 (*n* = 5)	5.0 ± 0.0 (*n* = 1)	2.7 ± 1.5 (*n* = 6)
Itching	2.7 ± 1.5 (*n* = 3)	1.7 ± 0.6 (*n* = 3)	4.3 ± 1.2 (*n* = 8)	3.5+ 0 0.7 (*n* = 2)	4.0 ± 1.8 (*n* = 6)	2.0 ± 1.0 (*n* = 3)
Burning	4.0 ± 1.4 (*n* = 2)	2.7 ± 0.6 (*n* = 3)	3.7 ± 3.1 (*n* = 3)	1.6 ± 0.9 (*n* = 5)	6.0 ± 1.4 (*n* = 5)	4.0 ± 1.4 (*n* = 5)
Prickling	3.0 ± 1.2 (*n* = 4)	NR	5.5 + 0.7 (*n* = 2)	3.0 ± 0.0 (*n* = 1)	4.0 ± 0.0 (*n* = 1)	NR
Poking	6.0 ± 0.0 (*n* = 1)	NR	NR	NR	2.0 ± 0.0 (*n* = 1)	4.0 ± 0.0 (*n* = 1)
Pins/Needles	NR	NR	3.0 ± 0.0 (*n* = 1)	NR	6.0 ± 0.0 (*n* = 1)	2.0 ± 1.4 (*n* = 2)
Stinging	NR	1.5 ± 0.7 (*n* = 2)	NR	2.0 ± 0.0 (*n* = 2)	NR	NR
Pinching	NR	NR	4.0 ± 0.0 (*n* = 1)	NR	NR	NR
**Blinding**						
Guessed sham	**60%**	**70%**	20%	20%	20%	10%
Guessed 2 mA	40%	30%	**50%**	**60%**	40%	40%
Guessed 4 mA	0%	0%	30%	20%	**40%**	**50%**

Sensations were reported on a 10-point Likert-type scale (1 = “barely perceptible” and 10 = “most I could possible stand”). Blinding results are percent guessed (correct guesses bolded). NR = none reported.

## References

[1] Nitsche M.A., Paulus W. (2000). Excitability changes induced in the human motor cortex by weak transcranial direct current stimulation. J. Physiol..

[2] Lefaucheur J.P., Antal A., Ayache S.S., Benninger D.H., Brunelin J., Cogiamanian F., Cotelli M., De Ridder D., Ferrucci R., Langguth B. (2017). Evidence-based guidelines on the therapeutic use of transcranial direct current stimulation (tDCS). Clin. Neurophysiol..

[3] Horvath J.C., Forte J.D., Carter O. (2015). Evidence that transcranial direct current stimulation (tDCS) generates little-to-no reliable neurophysiologic effect beyond MEP amplitude modulation in healthy human subjects: A systematic review. Neuropsychologia.

[4] Voroslakos M., Takeuchi Y., Brinyiczki K., Zombori T., Oliva A., Fernandez-Ruiz A., Kozak G., Kincses Z.T., Ivanyi B., Buzsaki G. (2018). Direct effects of transcranial electric stimulation on brain circuits in rats and humans. Nat. Commun..

[5] Kuo M.F., Paulus W., Nitsche M.A. (2006). Sex differences in cortical neuroplasticity in humans. Neuroreport.

[6] Russell M., Goodman T., Wang Q., Groshong B., Lyeth B.G. (2014). Gender Differences in Current Received during Transcranial Electrical Stimulation. Front. Psychiatry.

[7] Chaieb L., Antal A., Paulus W. (2008). Gender-specific modulation of short-term neuroplasticity in the visual cortex induced by transcranial direct current stimulation. Vis. Neurosci..

[8] Krause B., Cohen Kadosh R. (2014). Not all brains are created equal: The relevance of individual differences in responsiveness to transcranial electrical stimulation. Front. Syst. Neurosci..

[9] Meiron O., Lavidor M. (2013). Unilateral prefrontal direct current stimulation effects are modulated by working memory load and gender. Brain Stimul..

[10] de Tommaso M., Invitto S., Ricci K., Lucchese V., Delussi M., Quattromini P., Bettocchi S., Pinto V., Lancioni G., Livrea P. (2014). Effects of anodal TDCS stimulation of left parietal cortex on visual spatial attention tasks in men and women across menstrual cycle. Neurosci. Lett..

[11] Lapenta O.M., Fregni F., Oberman L.M., Boggio P.S. (2012). Bilateral temporal cortex transcranial direct current stimulation worsens male performance in a multisensory integration task. Neurosci. Lett..

[12] Boggio P.S., Rocha R.R., da Silva M.T., Fregni F. (2008). Differential modulatory effects of transcranial direct current stimulation on a facial expression go-no-go task in males and females. Neurosci. Lett..

[13] Rudroff T., Kindred J.H., Ketelhut N.B. (2016). Fatigue in Multiple Sclerosis: Misconceptions and Future Research Directions. Front. Neurol..

[14] Workman C.D., Kamholz J., Rudroff T. (2019). The Tolerability and Efficacy of 4 mA Transcranial Direct Current Stimulation on Leg Muscle Fatigability. Brain Sci..

[15] Workman C.D., Kamholz J., Rudroff T. (2020). Increased leg muscle fatigability during 2 mA and 4 mA transcranial direct current stimulation over the left motor cortex. Exp. Brain Res..

[16] Angius L., Pascual-Leone A., Santarnecchi E. (2018). Brain stimulation and physical performance. Prog. Brain Res..

[17] Cancelli A., Cottone C., Giordani A., Migliore S., Lupoi D., Porcaro C., Mirabella M., Rossini P.M., Filippi M.M., Tecchio F. (2018). Personalized, bilateral whole-body somatosensory cortex stimulation to relieve fatigue in multiple sclerosis. Mult. Scler..

[18] Ferrucci R., Priori A. (2014). Transcranial cerebellar direct current stimulation (tcDCS): Motor control, cognition, learning and emotions. Neuroimage.

[19] Lefaucheur J.P., Chalah M.A., Mhalla A., Palm U., Ayache S.S., Mylius V. (2017). The treatment of fatigue by non-invasive brain stimulation. Neurophysiol. Clin..

[20] Proessl F., Poston B., Rudroff T. (2018). Does a single application of anodal tDCS improve knee extensor fatigability in people with multiple sclerosis?. Brain Stimul..

[21] Tecchio F., Cancelli A., Cottone C., Zito G., Pasqualetti P., Ghazaryan A., Rossini P.M., Filippi M.M. (2014). Multiple sclerosis fatigue relief by bilateral somatosensory cortex neuromodulation. J. Neurol..

[22] Workman C.D., Kamholz J., Rudroff T. (2020). Transcranial direct current stimulation (tDCS) for the treatment of a Multiple Sclerosis symptom cluster. Brain Stimul..

[23] Gleeson N.P., Mercer T.H. (1992). Reproducibility of isokinetic leg strength and endurance characteristics of adult men and women. Eur. J. Appl. Physiol. Occup. Physiol..

[24] Jang H., Lee J.Y., Lee K.I., Park K.M. (2017). Are there differences in brain morphology according to handedness?. Brain Behav..

[25] Saenz A., Avellanet M., Hijos E., Chaler J., Garreta R., Pujol E., Sandoval B., Buen C., Farreny A. (2010). Knee isokinetic test-retest: A multicentre knee isokinetic test-retest study of a fatigue protocol. Eur. J. Phys. Rehabil. Med..

[26] Ciccone A.B., Deckert J.A., Schlabs C.R., Tilden M.J., Herda T.J., Gallagher P.M., Weir J.P. (2019). Transcranial Direct Current Stimulation of the Temporal Lobe Does Not Affect High-Intensity Work Capacity. J. Strength Cond. Res..

[27] Hameau S., Bensmail D., Roche N., Zory R. (2018). Adaptations of fatigue and fatigability after a short intensive, combined rehabilitation program in patients with multiple sclerosis. J. Rehabil. Med..

[28] Lambert C.P., Archer R.L., Evans W.J. (2001). Muscle strength and fatigue during isokinetic exercise in individuals with multiple sclerosis. Med. Sci. Sports Exerc..

[29] Mackey C.S., Thiele R.M., Conchola E.C., DeFreitas J.M. (2018). Comparison of fatigue responses and rapid force characteristics between explosive- and traditional-resistance-trained males. Eur. J. Appl. Physiol..

[30] Thorstensson A., Karlsson J. (1976). Fatiguability and fibre composition of human skeletal muscle. Acta Physiol. Scand..

[31] Klem G.H., Luders H.O., Jasper H.H., Elger C. (1999). The ten-twenty electrode system of the International Federation. The International Federation of Clinical Neurophysiology. Electroencephalogr. Clin. Neurophysiol. Suppl..

[32] Schambra H.M., Abe M., Luckenbaugh D.A., Reis J., Krakauer J.W., Cohen L.G. (2011). Probing for hemispheric specialization for motor skill learning: A transcranial direct current stimulation study. J. Neurophysiol..

[33] Jayaram G., Stinear J.W. (2009). The effects of transcranial stimulation on paretic lower limb motor excitability during walking. J. Clin. Neurophysiol..

[34] Foerster A.S., Rezaee Z., Paulus W., Nitsche M.A., Dutta A. (2018). Effects of Cathode Location and the Size of Anode on Anodal Transcranial Direct Current Stimulation Over the Leg Motor Area in Healthy Humans. Front. Neurosci..

[35] DaSilva A.F., Volz M.S., Bikson M., Fregni F. (2011). Electrode positioning and montage in transcranial direct current stimulation. J. Vis. Exp..

[36] Nitsche M.A., Cohen L.G., Wassermann E.M., Priori A., Lang N., Antal A., Paulus W., Hummel F., Boggio P.S., Fregni F. (2008). Transcranial direct current stimulation: State of the art 2008. Brain Stimul..

[37] Alix-Fages C., Romero-Arenas S., Castro-Alonso M., Colomer-Poveda D., Rio-Rodriguez D., Jerez-Martinez A., Fernandez-Del-Olmo M., Marquez G. (2019). Short-Term Effects of Anodal Transcranial Direct Current Stimulation on Endurance and Maximal Force Production. A Systematic Review and Meta-Analysis. J. Clin. Med..

[38] Stagg C.J., Jayaram G., Pastor D., Kincses Z.T., Matthews P.M., Johansen-Berg H. (2011). Polarity and timing-dependent effects of transcranial direct current stimulation in explicit motor learning. Neuropsychologia.

[39] Ammann C., Spampinato D., Marquez-Ruiz J. (2016). Modulating Motor Learning through Transcranial Direct-Current Stimulation: An Integrative View. Front. Psychol..

[40] Martin D.M., Liu R., Alonzo A., Green M., Loo C.K. (2014). Use of transcranial direct current stimulation (tDCS) to enhance cognitive training: Effect of timing of stimulation. Exp. Brain Res..

[41] Stagg C.J., Lin R.L., Mezue M., Segerdahl A., Kong Y., Xie J., Tracey I. (2013). Widespread modulation of cerebral perfusion induced during and after transcranial direct current stimulation applied to the left dorsolateral prefrontal cortex. J. Neurosci..

[42] Aparicio L.V.M., Guarienti F., Razza L.B., Carvalho A.F., Fregni F., Brunoni A.R. (2016). A Systematic Review on the Acceptability and Tolerability of Transcranial Direct Current Stimulation Treatment in Neuropsychiatry Trials. Brain Stimul..

[43] Gur H., Akova B., Punduk Z., Kucukoglu S. (1999). Effects of age on the reciprocal peak torque ratios during knee muscle contractions in elite soccer players. Scand. J. Med. Sci. Sports.

[44] Schulte-Geers C., Obert M., Schilling R.L., Harth S., Traupe H., Gizewski E.R., Verhoff M.A. (2011). Age and gender-dependent bone density changes of the human skull disclosed by high-resolution flat-panel computed tomography. Int. J. Legal Med..

[45] Workman C.D., Fietsam A.C., Uc E.Y., Rudroff T. (2020). Cerebellar Transcranial Direct Current Stimulation in People with Parkinson’s Disease: A Pilot Study. Brain Sci..

[46] Chhatbar P.Y., Kautz S.A., Takacs I., Rowland N.C., Revuelta G.J., George M.S., Bikson M., Feng W. (2018). Evidence of transcranial direct current stimulation-generated electric fields at subthalamic level in human brain in vivo. Brain Stimul..

[47] Hunter S.K. (2016). The Relevance of Sex Differences in Performance Fatigability. Med. Sci. Sports Exerc..

[48] Ditor D.S., Hicks A.L. (2000). The effect of age and gender on the relative fatigability of the human adductor pollicis muscle. Can. J. Physiol. Pharmacol..

[49] Russ D.W., Lanza I.R., Rothman D., Kent-Braun J.A. (2005). Sex differences in glycolysis during brief, intense isometric contractions. Muscle Nerve.

[50] Enoka R.M., Stuart D.G. (1992). Neurobiology of muscle fatigue. J. Appl. Physiol..

[51] Hunter S.K. (2009). Sex differences and mechanisms of task-specific muscle fatigue. Exerc. Sport Sci. Rev..

[52] Yoon T., Doyel R., Widule C., Hunter S.K. (2015). Sex differences with aging in the fatigability of dynamic contractions. Exp. Gerontol..

[53] Senefeld J., Yoon T., Bement M.H., Hunter S.K. (2013). Fatigue and recovery from dynamic contractions in men and women differ for arm and leg muscles. Muscle Nerve.

[54] Barclay C.J., Constable J.K., Gibbs C.L. (1993). Energetics of fast- and slow-twitch muscles of the mouse. J. Physiol..

[55] Fertonani A., Ferrari C., Miniussi C. (2015). What do you feel if I apply transcranial electric stimulation? Safety, sensations and secondary induced effects. Clin. Neurophysiol..

[56] Kessler S.K., Turkeltaub P.E., Benson J.G., Hamilton R.H. (2012). Differences in the experience of active and sham transcranial direct current stimulation. Brain Stimul..

[57] Weightman M., Brittain J.-S., Punt D., Miall R.C., Jenkinson N. (2020). Targeted tDCS selectively improves motor adaptation with the proximal and distal upper limb. Brain Stimul..

[58] Russo R., Wallace D., Fitzgerald P.B., Cooper N.R. (2013). Perception of comfort during active and sham transcranial direct current stimulation: A double blind study. Brain Stimul..

[59] Kawata M. (1995). Roles of steroid hormones and their receptors in structural organization in the nervous system. Neurosci. Res..

[60] Lambert J.J., Belelli D., Hill-Venning C., Peters J.A. (1995). Neurosteroids and GABAA receptor function. Trends Pharmacol. Sci..

[61] Mellon S.H. (1994). Neurosteroids: Biochemistry, modes of action, and clinical relevance. J. Clin. Endocrinol. Metab..

[62] Rupprecht R., Holsboer F. (1999). Neuroactive steroids: Mechanisms of action and neuropsychopharmacological perspectives. Trends Neurosci..

[63] Inghilleri M., Conte A., Curra A., Frasca V., Lorenzano C., Berardelli A. (2004). Ovarian hormones and cortical excitability. An rTMS study in humans. Clin. Neurophysiol..

[64] O’Connell N.E., Cossar J., Marston L., Wand B.M., Bunce D., Moseley G.L., De Souza L.H. (2012). Rethinking clinical trials of transcranial direct current stimulation: Participant and assessor blinding is inadequate at intensities of 2 mA. PLoS ONE.

